# The DBCLS BioHackathon: standardization and interoperability for bioinformatics web services and workflows. The DBCLS BioHackathon Consortium*

**DOI:** 10.1186/2041-1480-1-8

**Published:** 2010-08-21

**Authors:** Toshiaki Katayama, Kazuharu Arakawa, Mitsuteru Nakao, Keiichiro Ono, Kiyoko F Aoki-Kinoshita, Yasunori Yamamoto, Atsuko Yamaguchi, Shuichi Kawashima, Hong-Woo Chun, Jan Aerts, Bruno Aranda, Lord Hendrix Barboza, Raoul JP Bonnal, Richard Bruskiewich, Jan C Bryne, José M Fernández, Akira Funahashi, Paul MK Gordon, Naohisa Goto, Andreas Groscurth, Alex Gutteridge, Richard Holland, Yoshinobu Kano, Edward A Kawas, Arnaud Kerhornou, Eri Kibukawa, Akira R Kinjo, Michael Kuhn, Hilmar Lapp, Heikki Lehvaslaiho, Hiroyuki Nakamura, Yasukazu Nakamura, Tatsuya Nishizawa, Chikashi Nobata, Tamotsu Noguchi, Thomas M Oinn, Shinobu Okamoto, Stuart Owen, Evangelos Pafilis, Matthew Pocock, Pjotr Prins, René Ranzinger, Florian Reisinger, Lukasz Salwinski, Mark Schreiber, Martin Senger, Yasumasa Shigemoto, Daron M Standley, Hideaki Sugawara, Toshiyuki Tashiro, Oswaldo Trelles, Rutger A Vos, Mark D Wilkinson, William York, Christian M Zmasek, Kiyoshi Asai, Toshihisa Takagi

**Affiliations:** 1 Database Center for Life Science, Research Organization of Information and Systems, 2-11-16 Yayoi, Bunkyo-ku, Tokyo, 113-0032, Japan

## Abstract

Web services have become a key technology for bioinformatics, since life science databases are globally decentralized and the exponential increase in the amount of available data demands for efficient systems without the need to transfer entire databases for every step of an analysis. However, various incompatibilities among database resources and analysis services make it difficult to connect and integrate these into interoperable workflows. To resolve this situation, we invited domain specialists from web service providers, client software developers, Open Bio* projects, the BioMoby project and researchers of emerging areas where a standard exchange data format is not well established, for an intensive collaboration entitled the BioHackathon 2008. The meeting was hosted by the Database Center for Life Science (DBCLS) and Computational Biology Research Center (CBRC) and was held in Tokyo from February 11th to 15th, 2008. In this report we highlight the work accomplished and the common issues arisen from this event, including the standardization of data exchange formats and services in the emerging fields of glycoinformatics, biological interaction networks, text mining, and phyloinformatics. In addition, common shared object development based on BioSQL, as well as technical challenges in large data management, asynchronous services, and security are discussed. Consequently, we improved interoperability of web services in several fields, however, further cooperation among major database centers and continued collaborative efforts between service providers and software developers are still necessary for an effective advance in bioinformatics web service technologies.

## Introduction

Web services are software systems designed to be manipulated remotely over a network, often through web-based application programming interfaces (APIs). Through web services, users can take advantage of the latest maintained data and computational resources of remote service providers via a thin client. Web services are increasingly being adopted in the field of bioinformatics as an effective means for data and software access, especially in light of the rapid accumulation of large amounts of information for the life sciences [1]. Most of the major bioinformatics centers, including the National Center for Biotechnology Information (NCBI) in the US [2], the European Bioinformatics Institute (EBI) in the UK [3], and the DNA Data Bank of Japan (DDBJ) [4]/Kyoto Encyclopedia of Genes and Genomes (KEGG) [5]/Protein Data Bank Japan (PDBj) [6] in Japan, provide web service interfaces to their databases and computational resources. Since the web service model is based on open standards, these services are designed and expected to be interoperable [7]. However, many of the services currently available use their own data type definitions and naming conventions, resulting in a lack of interoperability that makes it harder for end users and developers to utilize these services for the creation of biological analysis workflows [8]. Moreover, these services are often not easily usable from programs written in specific computer languages, despite the language-independent specification of web services themselves. Some of the main reasons for that are the use of functionality not supported in a particular web service software implementation, and the lack of compliance with the SOAP/WSDL specification in a programming language's web service libraries.

To overcome this situation and to assure interoperability between web services for biology, standardization of exchangeable data types and adoption of compatible interfaces to each service are essential. As a pilot study, the BioMoby project has tried to solve these problems by defining ontologies for data types and methods used in its services, and by providing a centralized repository for service discovery. Additionally, Moby client software exists to allow interconnections of multiple web services [9,10]. However, there are still many major service providers that are not yet covered by the BioMoby framework and the Open Bio* libraries such as BioPerl [11], BioPython [12], BioRuby [13], and BioJava [14] have independently implemented access modules for some of these services [15].

To address these issues, we organized the BioHackathon 2008 [16], an international workshop sponsored by two Japanese bioinformatics centers, the Database Center for Life Science (DBCLS) [17] and the Computational Biology Research Center (CBRC) [18], focusing on the standardization and interoperability of web services. The meeting consisted of two parts: the first day was dedicated to keynote presentations and "open space" style discussions to identify current problems and to decide on strategies for possible solutions in each subgroup. The remaining four days were allotted for an intensive software coding event. Standardization and interoperability of web services were discussed by experts invited from four different domains: 1) web service providers, 2) Open Bio* developers, 3) workflow client developers, and 4) BioMoby project developers. Providers of independent web services were encouraged to address standardization and service integration, and were also asked to implement (and hence increase the number of) SOAP-compliant services for analysis tools and databases. Open Bio* developers focused on the utilization of as many bioinformatics web services as possible in four major computer languages (Perl, Python, Ruby, and Java), and collaborated to create compatible data models for common biological objects such as sequences and phylogenetic trees within the Open Bio* libraries. Workflow client developers were challenged to create and execute bioinformatics workflows combining various web service resources, and BioMoby project developers explored the best solution to define standard objects and ontologies in bioinformatics web services. In the following sections, we review the outcomes of standardization and interoperability discussions as well as the future challenges and directions of web services for bioinformatics that were highlighted in this workshop.

## Web service technologies

Bioinformatics web services can be categorized into two major functional groups: data access and analysis. Access to public database repositories is obviously fundamental to bioinformatics research, and various systems have been developed for this purpose, such as Entrez at NCBI, Sequence Retrieval System (SRS) and EB-eye at EBI [19], Distributed Annotation System (DAS) [20], All-round Retrieval for Sequence and Annotation (ARSA) and getentry at DDBJ [21], DBGET at KEGG [22], and XML-based Protein Structure Search Service (xPSSS) at PDBj [6]. These services provide programmable means for text-based keyword search and entry retrieval from their backend databases, which mostly consist of static entries written either in semi-structured text or XML. As each entry has a unique identifier it is generally assignable to a URI (Uniform Resource Identifiers).

The other group of services provides a variety of methods that require a certain amount of computation by implementing various algorithms, and they sometimes have complex input or output data structures. A typical example is a BLAST search, which needs a nucleic or amino acid sequence, as well as numerous optional arguments in order to find homologous sequences from a specified database using a dynamic programming algorithm. Services in this group sometimes require a large amount of computation time, including those providing certain functionalities of the European Molecular Biology Open Software Suite (EMBOSS) [23], 3 D structural analysis of proteins, and data mapping on biochemical pathways.

Historically, the term web services was associated with SOAP (Simple Object Access Protocol), a protocol that transfers messages in a SOAP XML envelope between a server and a client, usually over the Hypertext Transfer Protocol, HTTP [24]. SOAP services have several accessibility advantages, including an open standard that is independent from computer programming languages, and the use of the HTTP protocol which is usually not filtered by firewalls (SOAP services can therefore be accessed even from institutions having very strict security policies for Internet access). Since all SOAP messages are XML documents and the format of the messages are known in advance from the service description (see below), it is possible to use XML binding to seamlessly convert the messages to language-specific objects and thus avoid any custom-programmed parsing. XML binding is often leveraged by SOAP libraries to provide a programmatic interface to a web service similar to an object oriented API. Operations provided by SOAP services can consume several arguments, thus a service that requires a number of parameters can easily be utilized as an API, as if the method were a function call for a local library of a given programming language.

For the purpose of service description, SOAP services usually come with a Web Services Description Language (WSDL) [25] file. A WSDL file is an XML formatted document that is consumed by a SOAP/WSDL library to allow automatic construction of a set of functions for the client program. In addition to the list of methods, WSDL contains descriptions for each method, including the types and numbers of input arguments as well as those of output data. WSDL is also capable of describing complex data models that combine basic data types into nested data objects. In this way, SOAP services can accept various kinds of complex biological objects, such as a protein sequence entry accompanied by several annotation properties like the identifier, description, and source organism.

Recently, another kind of web service model named REST (Representational State Transfer) has rapidly gained popularity as an effective alternative approach to SOAP-based web services [1]. REST is an approach whereby an online service is decomposed into uniquely identifiable, stateless resources that can be called as a URL and return the relevant data in any format. Typically, many bioinformatics database services return entries in a text-based flatfile format upon REST calls. The strength of REST is in its simplicity. Since REST is built on top of HTTP requests, there is no need for supporting libraries, unlike SOAP/WSDL services. RESTful URLs are also highly suitable for permanent resource mapping, such as that between a database entry and a unique URI; therefore, biological web services that provide data access should ideally be exposed as simple REST services. On the other hand, REST is less appropriate for services that require complex input with multiple numbers of parameters, or for time-consuming and therefore asynchronous and stateful services. For those, SOAP/WSDL-based services are still more suitable.

WSDL description *per se *is not enough for the immediate construction of biological workflows as multiple cascading web services, because of inconsistent data types defined by each service provider, sometimes even for essentially identical objects. Therefore, in most cases output of one service cannot be passed to another service as its input without appropriate conversion of data types or formats. Furthermore, services should also be discoverable by the object models they share so they can be linked in the construction of workflows. To this end, a centralized registry to discover appropriate services according to a given set of data types has become essential for web service interoperability. The BioMoby project has pioneered this task by providing MobyCentral, which serves as a central repository for BioMoby compatible web services [9]. Service developers are encouraged to register their own service to the repository with a description of the service using the BioMoby ontologies that classify the semantic attributes of the method including the input and output data types. Metadata and ontologies for service description and discovery discussed during the BioHackathon are listed in Table 1.

**Table 1:** Required metadata for service description and discovery.

Required metadata for service description	
author contact	
authority identification	
service version	
software title or nature of algorithm (myGrid Task ontology)	
software version	
bandwidth and/or number of requests per minute	
example input	
example output and/or REGEXP to test output	
some description of error-handling capacity	
sync/async	
nature of underlying data	
organism	
biological nature of data (DNA/RNA/Protein, experimental methods or platform)	
input parameters and purpose of each	
output parameters and purpose of each	
usage/license restrictions	
authentication (whether required or not)	
usage statistics (as per service provider)	
usage statistics (as per third party commentary)	
protocol (Moby, SOAP, REST, GET, POST, etc.)	
mirror servers	
	

**Ontologies that could provide the above metadata**	

myGrid Ontology	provides many of the annotation information elements listed above
Moby Object	provides an ontology of data-types
Moby Service	similar to myGrid's bioinformatics_task branch of the myGrid Ontology

To date, several applications that utilize BioMoby services have been developed, such as Taverna [26], Seahawk [27], MOWserv [8], and G-language Genome Analysis Environment (G-language GAE) [28]. Taverna is a software tool developed under the myGrid project [29], written in Java and equipped with a graphical user interface (GUI) for the construction of workflows by interconnecting existing web services. Users can start from an initial set of data pipelined to a service, where the input data is remotely analyzed, resulting in an output of different data types. This output becomes the input for the subsequent analysis step, for which appropriate services that consume this input data type can be looked up, for example, through MobyCentral. Iteration of this procedure leads to cascading services forming a bioinformatics workflow, which can be repeatedly utilized with different datasets. The strength of Taverna is in its support of many non-BioMoby services that can be utilized in concert with BioMoby-based services, and its customizability by enabling small Java plug-ins to be written, for example to connect two services requiring data format conversion.

Seahawk is another GUI software tool that invokes BioMoby services in a context-dependent manner, for example, by selecting an amino acid sequence in a website to use as input data, so that users can analyze data as they browse information on web pages.

MOWserv [30] is a web application that provides interactive analysis in a web browser. A web interface is dynamically generated for each BioMoby object and compatible service. MOWserv implements novel functionality to allow data persistence, user management, task scheduling and fault-tolerance capabilities. Therefore MOWserv allows monitoring of long and CPU-intensive tasks and automating the execution of complex workflows. Invocation of services can be traced in the web interface, including for later reference. An interesting aspect of MOWserv is that it has extended the BioMoby ontologies for objects and services through manual curation. This keeps ontologies clean enough, so that it greatly simplifies interoperability between services and helps in building workflows. Additionally, each service has been annotated with additional metadata that is used to provide a consistent help system.

G-language GAE [31] is a Perl based genome analysis workbench that provides an interactive command-line shell environment for analyses. During the BioHackathon 2008, the G-language Project team added support for BioMoby services that can be seamlessly integrated with BioPerl and G-language GAE functions into genome analysis workflows. Also, it became evident during the hackathon that there needs to be a standardized way to retrofit existing web services to BioMoby, and this work started on this using the World Wide Web Consortiums' new SAWSDL standard [32].

For many tasks custom programming is still needed, for example, to parse the results obtained from web services for further extraction of data, and to integrate with local analysis pipelines. One of these most time-saving ways to accomplish these tasks is by using the Open Bio* libraries, such as BioPerl, BioPython, BioRuby and BioJava. These libraries are being collaboratively developed as open source software by developers distributed all over the world, and they have the capability to manipulate numerous formats used in bioinformatics databases and applications. The Open Bio Foundation [33] has an important role in supporting these projects by providing hosting services for the code repository, mailing lists, and web sites to the community.

SOAP and REST have improved accessibility of bioinformatics web services, but standardization of metadata is required to increase their interoperability (Table 1). Although BioMoby has been contributing to it, many major services still have not adopted its formalities. This situation leaves end-users many cases where they have to make a code to construct a workflow. Even though some GUI applications or libraries of each programming language are provided to support it, there has not been a "total solution," yet (Table 2). Considering these circumstances, a web service to convert data formats would be needed to alleviate the end-users' tasks.

**Table 2:** Applications for bioinformatics web services.

Project	Description	GUI	Open source	Programming Language
BioMoby/MobyCentral	Framework/repository of the interoperable web services	-	o	Perl/Java
Taverna	Workflow construction tool to connect web services in a pipeline	o	o	Java (BeanShell script to extend)
Seahawk	Graphical interface to invoke appropriate BioMoby services	o	o	Java
MOWserv	Web application to handle BioMoby services in the grid environment	o	-	-
G-language GAE	Command line shell to access BioMoby and other web services	-	o	Perl
Open Bio*	Libraries including supports for bioinformatics web services	-	o	Perl/Python/Ruby/Java

## Standardization

Data types exchanged among bioinformatics web services should ideally follow commonly accepted standards in order to be interoperable without data format conversion. However, in emerging areas of bioinformatics such as protein interaction networks, glycoinformatics, phyloinformatics and text mining, several standard formats have been independently developed, and in many cases data have to be merged and integrated prior to analysis because they need to be collected from multiple repositories. Moreover, identifiers and controlled vocabularies employed in these separate repositories are often different even for identical physical entities. To address these issues, we have gathered a wide range of data providers in their respective areas to discuss obstacles and implement solutions towards interoperable services.

## Biological Interaction Network

In this hackathon representatives from three different service providers, the Database of Interacting Proteins (DIP) [34], the Search Tool for the Retrieval of Interacting Genes/Proteins (STRING) [35]/the Search Tool for Interactions of Chemicals (STITCH) [36], and IntAct [37,38], as well as representatives from two service consumer projects, Cytoscape [39] and CellDesigner [40], jointly discussed the most prominent issues. These included a standardized data format for interaction networks, a common API for interaction web services, and an ontology for molecular interaction data. The Systems Biology Markup Language (SBML) [41] or the Biological Pathways Exchange (BioPAX) [42] formats can be used to represent molecular pathways, but the Proteomics Standards Initiative-Molecular Interactions (PSI-MI) seems to be the stronger with experimental and interaction data while not being as computationally complex than the more flexible approach taken by BioPAX [43]. The group agreed to use PSI-MI 2.5 [44] as the standard exchange format (both XML-based MIF and tabulated MITAB), which is an existing format widely used in the biological interaction network analysis community. This also solved the problem of defining an interaction data ontology since PSI-MI is based on a well-defined ontology maintained by the Molecular Interaction workgroup of the HUPO Proteomics Standards Initiative (PSI) [45]. This allowed the design of a new protocol called PSICQUIC (PSI Common Query Interface). It is a simple API to search and retrieve PSI-MI-based datasets using either dedicated functions or a simple query language using the Apache Lucene syntax. In order to cope with the large amount of returned information, all PSCICQUIC queries can be paginated. A WSDL was created that can be used by the different resources to provide molecular interaction data programmatically, and a proof-of-concept of this approach was demonstrated. Based on this specification, ten PSICQUIC web services [46] including IntAct and iRefIndex [47] has been developed since then.

As a test client for PSICQUIC the group used the Cytoscape visualization platform. Since version 2.6, Cytoscape supports web services as external data sources. It is relatively easy to implement a client in Cytoscape because the data exchange format is based on PSI-MI (which is already supported by the software). The Cytoscape team is implementing a universal client for PSICQUIC, supporting network data integration and visualization from multiple data sources. The working group and the International Molecular Exchange (IMEx) consortium [48] members continue to work on this project and publish standard services along with the reference implementation of the PSICQUIC client.

## Glycoinformatics

The goal of the glycomics standards and interoperability group was to integrate emerging bioinformatics tools for glycobiology into the larger bioinformatics world, primarily by establishing a foundation for web services for the glycobiology community [49]. Bioinformatics for glycobiology is in its infancy, and the tools for identifying glycan structures, their biosynthetic mechanisms, and their biological functions are just being developed. The three participants in this group have taken active roles in developing these tools. A major obstacle in this endeavor is the difficulty in the non-ambiguous digital representation of complex glycans. This is due, in part, to the branched nature of glycans and the fact that the linkage between individual glycosyl residues can be complex. Several different successful representation protocols have been developed, including LINUCS (Linear Notation for Unique description of Carbohydrate Sequences) [50], and KCF (KEGG Chemical Function) [51]. Recently, it became clear that interoperability of the various databases and web services for glycobiology depends on a data exchange standard, which led to the development of GLYDE-II as a collaborative effort [52]. GLYDE-II is now almost completely functional, providing a key element for interoperability in glycoinformatics.

Further advancement in this area demands robust protocols for web service discovery and composition of web processes. The BioHackathon was a unique opportunity to get developers of glycoinformatics together to explore possibilities for this purpose. The immediate goal of the group was to develop a prototype workflow that integrates web services provided by the groups of the three represented glycoinformatics groups. This served as a test-bed and model for future integration efforts. There was a debate as to whether glycan structures should be integrated with the existing data types or to be defined separately. There was also a question as to whether the formats for these data types, such as the GLYDE-II XML formats, LINUCS and KCF, should be separate from other biomolecule sequence formats. In the end, it was decided that it would be easier to simply create a single GlycomicsObject data type in the BioMoby ontology from which all other data types would be extended. Using this consensus ontology, the three participants each provided web services that could communicate with one another, resulting in a single workflow. This workflow consisted of an input glycan structure in LINUCS format, and a search for glycans with similar structures in the RINGS (Resource For Informatics Of Glycans) database returning structures as KEGG GLYCAN IDs. The IDs were subsequently transformed to the corresponding GLYDE-II format data, which passed to another web service to output the structure's image in SVG format.

## Phyloinformatics

In the field of phyloinformatics, existing approaches to integrate data and services into workflows are highly specific to the integration platform (e.g. CIPRES, BioPerl, Bio::Phylo, Kepler) [53,54], and thus not immediately reusable as web services. In order to achieve an interoperable standard for the construction of generic web services, an agreement on the representation of the basic required objects for phylogenetic or phylogenomic analysis is necessary.

As a starting point, the group revisited the representation of phylogenetic trees and annotation (or metadata) often associated with nodes, branches, or the tree as a whole. For example, the branches of a phylogenetic tree can have length values or be associated with metrics indicating support, such as bootstrap value or posterior probability. Tree nodes might be associated with taxonomic information in the case of species trees. Nodes in gene trees may in addition be linked to gene names or identifiers and other gene or genome annotation. Beyond the more standard metadata elements, there is a large and increasing variety of data and annotation that is being associated with phylogenetic tree nodes in research applications, ranging from biogeographical data (latitude/longitude) to host species (for analyzing host/parasite co-evolution) to functional gene attributes (GO terms, gene expression data). Compared with plain text formats, the representation of such metadata according to an XML schema (as in phyloXML [55] and NeXML [56]) enables strict syntax validation and provides a standard framework for the integration of ontologies to describe the meaning of data and metadata, yet still allows the flexibility conferred by simple attribute/value pairs that can accommodate an unlimited number of metadata elements including new elements arising from new research approaches.

Tree reconciliation is another important class of problems for which standardization is a prerequisite for preparing such operations for wider adoption in web services. Specifically, the tips in a phylogenetic tree depict Operational Taxonomic Units (OTUs), which may stand for a species as represented by one or more specimens, or for one or more molecular sequences which belong to genes of a specific taxon. Reconciling trees involves matching tips in one tree to those in another where the trees may use different OTUs, or different conventions for labeling OTUs. For example, to infer gene duplication events from reconciling gene trees with species trees, the gene tree OTUs must be matched with the tips in the species tree, which requires identification of the canonical species to be unequivocal for both kinds of trees. A similar problem is encountered for applications that need to find trees in a database of trees, for example trees with nodes matching gene or species names, regardless of which kind of molecular sequence the trees of interest have been built from. A standardized encoding mechanism for OTUs would aid greatly towards exposing and consuming such operations in a consistent and predictable manner as web services.

Analysis-based web services for phyloinformatics applications typically require multiple types of data as input, some of which may be large, such as a distance or character matrix and one or many trees in respective order. While there are exchange formats that would allow marshalling of multiple data types in a single attachment or message body (e.g. a chunk of NEXUS [57] or NeXML [56]), this is often not desirable in web services due to network interruptions and bandwidth limits, and large parameter values may also easily exceed the capacity of URL-based requests to a RESTful web service. Alternatives that can solve some of these problems include passing parameters 'by reference' as globally unique identifiers (such as LSIDs [58]) rather than by value, and the accumulate-and-fire paradigm. The latter allows the calling agent to submit one parameter value at a time to accumulate at the service provider until the parameter list is complete, which would trigger execution of the service. As web services, and especially RESTful services are typically layered on top of the stateless HTTP protocol, supporting this calling paradigm would require additional mechanisms to maintain state between invocations. Hence, the conclusion reached at the BioHackathon was that such combinations of data would best be submitted as multiple parameters in a single request, but using the POST method of the HTTP protocol. A summary of input/output data types for phyloinformatic web services is provided in Table 3. Based on these considerations during the BioHackathon, a specification for RESTful phyloinformatic web services was proposed following the meeting. This specification, called PhyloWS [59], has been further developed at the Database Interoperability Hackathon [60] and onwards. At its most basic compliance level, the specification provides a simple API for assigning unique URLs to phylogenetic data objects (such as phylogenetic trees and character state matrices) and for retrieving them in various serialization formats using query string arguments. Prototype implementations of this compliance level have been created for TimeTree [61] and for the Tree of Life Web Project [62]. In addition to simple lookup of phylogenetic data objects, the PhyloWS specification also allows for searching using Contextual Query Language [63], a specification developed by the US Library of Congress that facilitates separation of search predicates from the underlying data provider's schema. Compliance at this level is provided by TreeBASE [64].

**Table 3:** Input and output data types relevant for phyloinformatic web services.

**Inputs **- The input data types defined here do not imply pass-by-value, and could be passed as an identifier:	
One Tree	exactly one tree, which might function as a query topology, as an input for topology metric calculations, or as something for which associated data (matrices) and metadata might be retrieved
Pair of Trees	exactly two trees, for tree reconciliation (e.g. duplication inference) or for tree-to-tree distance calculations
Set of Trees	input for consensus calculations, or as query topologies
One OTU	exactly one OTU for which associated data (trees or matrices that contain it) and metadata might be retrieved
Pair of OTUs	exactly two OTUs, as input for topological queries (MRCA) and calculations (patristic distance)
Set of OTUs	input for topological queries (MRCA) and for trees or matrices that contain them, and metadata is retrieved
One Node	input for tree traversal operations (parent, children) and for which metadata might be retrieved
Pair of Nodes	input for topological queries (MRCA) and calculations (patristic distance)
Set of Nodes	input for topological queries (MRCA)
One Character	exactly one character (matrix column) for which calculations are performed (variability) and metadata is retrieved
Set of Characters	input as filter predicate, to retrieve OTUs that contain recorded states for the characters
One Character State Sequence	input for which metadata is retrieved
Pair of Character State Sequences	input for pairwise alignments, as input to calculate pairwise divergence
Set of Character State Sequences	input for multiple sequence alignment
Character State Matrix	input for inference (of one tree or set of trees), for calculations (average sequence divergence) and metadata retrieval
	

**Outputs **- In addition to the mirroring the inputs described above, some 'primitives' may be required:	

Int	an integer, for things such as topology metrics (node counts) tree-to-tree distances (in branch moves) node distances (in number of nodes in between), character state counts, sequence divergence (substitution counts, site counts)
Float	a floating point value, for topology metrics (balance, stemminess, resolution) tree-to-tree distances (symmetric difference), patristic distance, sequence divergence
String	for metadata, e.g. descriptions
Stringvector	for metadata, e.g. a set of tags

## Text-mining

Natural Language Processing (NLP) technology has greatly improved in recent years, and enables us to syntactically analyze huge amounts of text data, such as the entire MEDLINE database. While useful biological knowledge can be extracted using this technology, all-in-one software package for easy utilization of state-of-the-art algorithms is still lacking. In addition, the existence of several similar applications with their own specific functions can make it difficult to readily apply NLP in everyday research. Typically, in order to extract biological knowledge from a large amount of text, a series of NLP tools are sequentially applied as follows: 1) a sentence splitter outputs one sentence per line from a given text, 2) a Part-of-Speech (POS) tagger outputs the set of pairs of POS tags and their corresponding word positions in the given sentence, 3) a named entity recognizer (NER) outputs the set of pairs of a domain-specific term such as a gene or protein name and their positions from the given POS-tagged sentence, 4) a deep parser outputs a syntactic tree that describes syntactic dependencies among words of the sentence from the POS with NER-tagged sentence, and finally, 5) an information extraction (IE) tool indicates some biological knowledge such as protein-protein-interactions. At each step, several research groups have developed tools for their own needs, and interoperability has correspondingly suffered.

The Unstructured Information Management Architecture (UIMA) [65] is an open framework developed to address this lack of interoperability. It was originally developed by IBM and is now an Apache project being widely used in the BioNLP community. UIMA provides a specification and a reference implementation for tools to transfer their inputs/outputs of unstructured data, such as text or images, easily and seamlessly in order to construct a workflow. However, UIMA itself is not enough to connect NLP tools in the field of biology and to realize the processes mentioned above. The BioNLP field is defining more detailed data types for biology and developing corresponding tools [66,67].

U-Compare [68] is an integrated NLP platform based on UIMA, developed as a collaborative project in the BioNLP community; U-Compare provides what UIMA is missing to be truly interoperable: It allows NLP users to easily combine and compare the existing applications, aids usability through visualizers, and assists developers. U-Compare also provides a large collection of ready-to-use interoperable tools and corpora, some of which are web services, and in fact U-Compare itself is distributed as a web application.

On the other hand, several components of NLP functions mentioned above are available independently over SOAP, such as NER, domain specific dictionary lookup, or abbreviation searching, as can be seen in services like Whatizit [69] and TerMine [70]. There are also databases of text-mined information that provide web service APIs such as iHOP [71], BioCreative [72] and Allie [73]. UIMA itself provides a SOAP interface available for any UIMA component. While BioNLP tools seem to be readily linked with other bioinformatics web services, the raw data generated by several BioNLP tools, such as a syntactic parse tree in an XML format, tend to be complex data structures, which requires the recipient web service to parse and interpret the data. This situation is essentially the same as for the local NLP services.

## BioSQL

The results obtained from web services will inevitably need to be manipulated locally. Ideally this can happen in a manner that can fully harness and is interoperable between the Bio* libraries, such as BioPerl, BioRuby, BioPython, and BioJava. The Bio* libraries, however, are based on different and independently developed DNA sequence models, and therefore there is no obvious or common way to share object types among these projects. Although the International Nucleotide Sequence Databases (INSD) defined a standard format for DNA sequence and its annotation [74], the format specification itself does not assure consistency and compatibility of data converted to it.

In response to these needs, the Bio* projects have started to collaborate to utilize the programming language-independent BioSQL data model [75] as the basis for an interoperable set of entities and operations defined on them for storing, querying, and manipulating richly annotated biological sequence objects. The BioSQL project was originally started in 2001 as a means to store and query a local copy of GenBank in relational format, and has since evolved as a relational model and persistence interface that aims to be interoperable between the Bio* libraries. The core model covers sequences, sequence features, sequence annotation, and a reference taxonomy as well as controlled vocabularies and ontologies. Though significant progress has been made, full semantic interoperability has not yet been achieved due to differences in the way the Bio* projects interpret and represent different kinds of sequence annotation and sequence features. An agreed upon definition of the semantic mapping from common rich sequence formats to a shared entity model such as BioSQL could be the cornerstone for standardizing sequence and annotation semantics across the Bio* libraries, and serve as a reference to many web service providers and consumers that use sequence data.

The opportunities for cross-project collaboration during the BioHackathon allowed the BioSQL group to put the finishing touches on the schema and release the 1.0 version of BioSQL shortly after the event. Previously each of the major Bio* library projects had already developed bindings of their respective object models to the BioSQL relational model. Some of them, in particular the bindings for BioJava and BioRuby, were significantly improved at the event, in particular in regard to the ability to round-trip sequences as truthfully as possible through load and retrieve cycles. Aside from these activities, the group implemented a proof-of-concept BioSQL web service interface powered by Enterprise Java Beans (EJBs). A version ready for use in a production environment will need further optimizations that allow clients to retrieve only those attributes of sequence or annotation objects that they actually need. For example, a client retrieving a whole chromosome sequence entry that has numerous types of annotation attached may only be interested in a small subsequence and correspondingly only the annotation pertaining to that part, and possibly only certain types of annotation. The mechanisms that facilitate this include lazy (on-demand) loading of data, and implementation of call-backs.

## Standardization promotes interoperability

We discussed data exchange formats in the fields of Interaction Network, Glycoinformatics, and Phyloinformatics, respectively, and have begun to develop web services using them (Table 4). These activities indicate that standardization of data exchange formats facilitates development of related web services. In addition, it has an advantage in enabling us to provide web services that have higher interoperability, and workflow development will be eased.

**Table 4:** Standardization of data exchange formats and web services.

Domain	Format	Service	Relevant technologies
Interaction Network	PSI-MI	PSICQUIC	DIP, STRING, STITCH, IntAct, Cytoscape, Cell Designer
Glycoinformatics	GlycomicsObject	BioMoby	GLYDE-II, LINUCS, KEGG GLYCAN (KCF), RINGS
Phyloinformatics	phyloXML, NeXML	PhyloWS	CIPRES, Kepler, BioPerl (Bio::Phylo), NEXUS
Text-mining	U-Compare type system	U-Compare	UIMA, Whatizit, TerMine, iHOP, Allie
BioSQL	BioSQL schema	-	BioPerl, BioRuby, BioPython, BioJava

## Technical challenges

In order for web services to be utilized in high-throughput bioinformatics research, several common technical challenges exist, including management of large data (which is especially demanding in light of the recent development of next-generation sequencers), asynchronous execution, and data security. Below we summarize the discussions regarding these challenges.

## Managing large data

Transfer of large amounts of data through web services is problematic not only because of performance issues, but also because long transmissions are more likely to be interrupted by sporadic drops in network connectivity and similar transient problems. Aside from the susceptibility to interruption, large data sets sent inside of a SOAP envelope (using Base64 encoding) must always be loaded into memory on both client and server side, and existing web service client stacks often do not handle large documents in a robust manner. One feasible workaround is to send many gigabytes of data as Message Transmission Optimization Mechanism (MTOM) SOAP attachments [76]. MTOM is based on MIME, and can be processed separately from the SOAP envelope. Another way is to avoid sending the data itself through SOAP and pass it instead by reference; for example, through the use of a URI (possibly LSID [58]) fetching the data is not only delayed until the last step before execution, but it can be further optimized at the end of the service provider through a BitTorrent peer-to-peer (P2P) download [77]. BioMoby has proposed a mechanism to allow parts of the Moby data payload to be references, in order to achieve efficient management of large data in their framework. To retain type safety and argument semantics, references can be typed, and by advertising the types in the MobyCentral metadata registry they can be made available to clients such as Taverna.

We note that creating a service which accepts or creates references is not actually technically difficult; instead, the difficulty is in the advertisement of this capability, specifically with technologies such as WSDL that have no way to identify the actual data types of the de-referenced input values. The challenges that any system must solve to support reference passing are therefore: 1) acceptance of input data passed to the service as a reference type, 2) allowing the client to specify the delivery type for any results, 3) ideally a mechanism where a naive (non-reference aware) client is able to use the service without modification and 4) some level of lifecycle management for results held in a delivery location.

## Asynchronous service invocation

Some web service transactions can potentially take a long time to complete, exceeding the timeout threshold of intermediate communication protocols such as HTTP. It is possible to overcome this problem by using an asynchronous invocation model, where a single logical transaction is implemented as multiple, short-lived transport-level transactions. As the web service by itself is stateless, a mechanism needs to be employed to keep state across transactions. For this purpose, BioMoby uses the Web Service Resource Framework (WSRF) [78], which is well supported by the WSRF::Lite library in Perl. However, the library in Java has not been updated to the latest specification at the time of the hackathon, and there is no implementation in Ruby, preventing the development of asynchronous BioMoby clients in this language. Furthermore, WSRF is ratified by the Organization for the Advancement of Structured Information Standards (OASIS) [79], and it provides only limited compatibility with WS-I [80]. Several major service providers also have implemented or are in the process of implementing asynchronous services in several different ways: DDBJ WABI [81], PDBj, INB MOWserv, EBI-EMBOSS (SoapLab), EMBRACE NoE [82], and InstantSOAP [83]. Most of them provide custom solutions such as the polling model using a job identifier, but the use of HTTP Cookies to maintain state, for example, cannot be utilized by the Python library and even requires a client side implementation in other computer languages.

The default solution for asynchronous services in the web service stack is to use callback operations. However, the majority of web services in the bioinformatics domain are unidirectional, and it cannot be generally expected that a client would have the possibility to expose an external service interface and to accept incoming calls from the server. Therefore, a solution based on polling, ideally accompanied by descriptions in WSDL, is more light-weight and suitable since it does not assume that a client also exposes a service interface. One implementation of a polling-based approach is the SoapLab asynchronous interface [84], which is based on the Life Science Analysis Engine (LSAE) specification [85]. WSDL 2.0 also provides promising solutions, extending WSDL web service description capabilities to the REST world.

## Security

To make web services secure, there are two different layers to be considered; one is the transport security level and the other is the protection of shared resources. At the BioHackathon, requirements for the latter case were discussed to define the minimal information that should be provided by the client in order for the service to know who is trying to use it. Using such information, security services may enforce access control policies at all levels to provide secure authentication and communication over an open network, including: 1) resource protection by restricting the availability of software and computational resources, 2) protection of restricted or proprietary data, and 3) scheduling for priority-based systems. An authorization service is desirable for dynamic access control and security management over federated resources. An example implementation of such a management system can be seen in MyProxy [86], an open source security credential (certificates and private keys) repository for grid computing environments. After registration the user connects to the grid service portal and creates delegated credentials on a MyProxy repository, where delegation is achieved by the use of so-called proxy credentials. The user then uses different services and workflows through the portal, and when a service is called, the user is authenticated through the proxy certificates managed by the MyProxy. Services can thus depend on a central authorization service to determine the access level, and services offering access to sensitive data can require additional authorization decision requests to some other authorization service that implements the appropriate data protection policies. Minimal information required for client authentication would include: 1) authorization levels of a user according to UNIX-like permissions, 2) standardized interchange protocols and formats, 3) authentication based on X509 digital certificates, a technology commonly used for secure website connections (https), 4) certificates managed through a Public Key Infrastructure (PKI), which deals with the secure creation, validation and revocation of certificates, 5) availability of all relevant web services in the case of running a workflow, 6) profiling of offer deployment based on user rights or roles.

## Workflow integration case study

**Figure 1:**
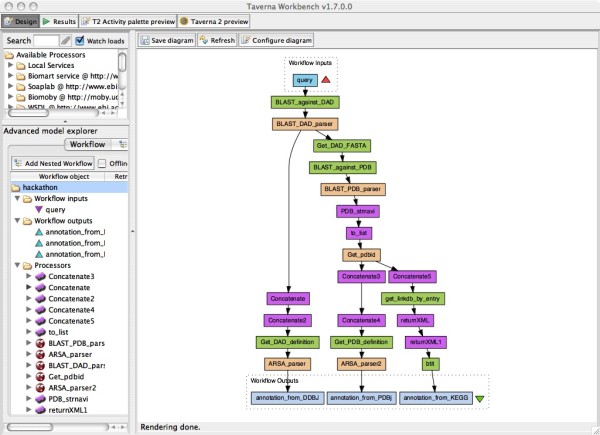
**Screenshot of Taverna workflow constructed as a case study that pipelines Japanese web services (DDBJ, PDBj and KEGG) to annotate a protein sequence by homology and structure**. Green boxes indicate the actual web services, and beige and purple boxes are local BeanShell script and Java shims that function as glue codes connecting the web services.

To explore the possibilities and limitations of service integration, we constructed a workflow in Taverna as a case study that pipelines web services from Japan-based providers (DDBJ, PDBj and KEGG) to annotate a protein sequence by homology and structure (Figure 1). In this workflow, given an unannotated protein sequence, 1) homologous sequences are searched using BLAST in the DDBJ DAD database, 2) corresponding annotations of the resulting homologs are retrieved from DDBJ, 3) when only hypothetical proteins are found, the BLAST search is extended to PDB, 4) homologs obtained in this way (both annotated or not) are sent to Structure-Navigator (structure search) at PDBj, and finally, 5) annotations are retrieved from PDBj and KEGG for entries with similar structures.

In the course of this evaluation, limitations in the current state of interoperability as well as possible challenges became apparent. Firstly, while the SOAP services provided by DDBJ and KEGG were readily usable, the required functionality in this workflow was missing in the SOAP-based API of PDBj, so that the REST interface was alternatively utilized after several necessary modifications. Therefore, although it is difficult to immediately standardize the data types, web services that are available can still be useful for users as long as they only require small modifications on the client side. Secondly, it turned out that Taverna at present does not support conditional branching, so the workflow had to be branched unconditionally. Thirdly, in most cases the output of one service could not be directly passed as the input of another service due to incompatibility of the data types, and small pieces of glue code were necessary for minor adjustments. Nonetheless, BeanShell scripts in Java provided by Taverna proved effective and useful for such formatting. To avoid or minimize glue code programming, it would be convenient if converters of data types were exposed themselves as a web services.

After the hackathon, PDBj has begun to provide a required API and the first issue is fixed. The second issue is essentially due to the Taverna software architecture/design, and end-users need to wait for another workflow management environment to be developed or write a code by themselves. To solve the third issue, DBCLS has started to develop a new web service called TogoWS [87], which enables end-users to seamlessly utilize web services provided by several heterogeneous providers. In addition, it provides a service to convert data formats to liberate end-users from making a glue-code when they construct a workflow. In our view, use of RDF as a data exchange format among major services will make construction of workflows even easier.

## Conclusions

Standardization efforts for exchange formats and service ontologies reached a certain level of agreement in the areas of biological interactions, phyloinformatics, glycoinformatics, and text-mining. However, there still remain several domains in biology where the basic exchange data types are not yet approved and relevant web services are not yet developed. Promoting standardization and interoperability efforts to these emerging areas are essential for integrative analysis, hence appropriate guidelines to develop standard web services are required. It is also very important that major bioinformatics database centers cooperate with each other towards this end. Accordingly, continued collaborative efforts among service providers, Open Bio* library developers, and workflow client developers are necessary for an effective advance in bioinformatics web service technologies.

Standardization and integration by their nature require intensive collaboration and coordination between independent projects and work groups. The gaps in the interoperability of web services therefore partly arise from the relative infrequency of opportunities for inter-project face-to-face discussion and collaboration. A highly intensive collaborative meeting with participants who have a wide variety of expertise therefore mitigates this problem, and a "hackathon" provides an effective and unique opportunity to make this happen [88]. Further increasing the interoperability of bioinformatics web services on a sustained basis would therefore likely benefit from regular BioHackathon events in the future.

## Competing interests

The authors declare that they have no competing interests.

## Authors' contributions

All authors attended the BioHackathon 2008. TK and KAr primarily wrote the manuscript. TK, YY, SK, MN, HC, AY, TNo, KAs and TTak organized the BioHackathon 2008. All authors read and approved the final manuscript.

## Authors' information

The DBCLS BioHackathon Consortium consists of more than 60 scientists from over 12 nations that attended the workshop that took place in Tokyo, sponsored by DBCLS and CBRC of Japan.
